# Regional variation in the use of catheter ablation for patients with arrhythmia in Japan

**DOI:** 10.1002/joa3.12455

**Published:** 2020-11-21

**Authors:** Takahiro Inoue, Hiroyo Kuwabara

**Affiliations:** ^1^ Healthcare Management Research Center Chiba University Hospital Chiba Japan

**Keywords:** arrhythmia, atrial fibrillation, catheter ablation, regional variation

## Abstract

**Background:**

Regional variation in the use of percutaneous coronary intervention (PCI), especially when performed as an elective procedure, was observed in a previous study. The use of a developing technology, catheter ablation (CA), was compared between regions in Japan.

**Methods and Results:**

The Diagnostic Procedure Combination data, which are publicly available, were used for the analysis. The number of CAs was summarized and the rates for CA and PCI were calculated based on the prefecture's population aged ≥40 years. A linear regression model was constructed to identify the factors associated with regional variation in the use of CA. The number of CAs performed per hospital consistently increased from 2009 to 2018. The mean rate of CA across Japan was 119 per 100 000 population aged ≥40 years in 2018. The highest CA rate was 166 per 100 000 and the lowest CA rate was 29 per 100 000 in 2018, while the highest and lowest PCI rates for angina per 100 000 were 361 and 88 in 2018, respectively. The significant factor associated with regional variation in the CA rate was the number of specialists.

**Conclusions:**

A wide regional variation was observed in the use of CA for patients with arrhythmia in Japan. Further research is needed to generate evidence of CA for decision‐making as a treatment option and to appropriately deploy this health service regardless of where patients live.

## Graphical abstract

Catheter ablation rate per 100 000 population aged ≥40 years in each prefecture in Japan. A wide regional variation was observed in the use of catheter ablation for patients with arrhythmia.

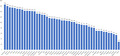

## 1 INTRODUCTION

Regional variation in health care has been well recognized in Japan as well as in many industrialized countries. The Japanese Ministry of Health, Labour and Welfare (MHLW) has reported regional variation in health‐care costs across Japan since 2009.^1^ The report in 2017 noted that the maximum‐to‐minimum ratio in the health‐care cost per person covered by the municipal national health insurance was 1.56‐fold, which has been relatively stable over the past 5 years; the report also suggested that cardiovascular disease was the most significant contributor to causing the difference in the use of health‐care resources and spending, in terms of disease perspective.^1^ In our previous study, regional variation in the use of percutaneous coronary intervention (PCI) was observed when the procedure was performed as an elective operation.^2^

Research to investigate causes and consequences of regional variation in health care has been actively conducted worldwide since Wennberg and Gittelson published their pivotal paper more than 40 years ago.^3^ They addressed unwarranted regional variation in the deployment of resources and utilization of health‐care services without clear patterns and reasons.^4^ In their subsequent studies of the area of cardiovascular disease, they observed regional variation in the use of coronary artery revascularization and the treatment of patients with myocardial infarction (MI) in the late 1990s.^5^, ^6^ From recent studies of the United States, regional variation is still observed in terms of the use of PCI.^7^ Furthermore, recent studies reported regional variation in newer technology and medical devices, such as transcatheter aortic valve implantation and cardiac implantable electrical devices.^8^, ^9^

As the aging population has grown, the prevalence of arrhythmia, including atrial fibrillation (AF), has increased.^10^ An Australian study reported that the provision of catheter ablation (CA) for AF has increased markedly during the past decade.^11^ It is important to assess whether access to this technology is secure in Japan. The objective of the present study is to investigate regional variation in the use of CA for patients with arrhythmia in Japan.

## 2 METHODS

### 2.1 Data source and study population

We obtained the data for the present study from the assessment report on the impact of implementing the Diagnostic Procedure Combination/Per‐Diem Payment System (DPC/PDPS) system through the website of the MHLW.^12^ DPC/PDPS is used as reimbursement scheme for the acute phase in‐patient service in Japan, and the DPC code is defined by the most resource‐consuming disease and its medical procedures during hospitalization. The assessment report contains the summary table of each DPC code by each hospital across Japan. We selected the DPC code specifying tachyarrhythmia (DPC code: 050070) as the most resource‐consuming disease and further focused on the code indicating CA as a medical procedure for the source of the study population. The data collected in 2018 were used to calculate the rates of CA per prefecture and the data collected from 2009 to 2018 were used to describe the underlying disease for CA and the number of CA performed per hospital in each year. In addition, we used patient‐level DPC data collected by 90 hospitals from 30 prefectures to describe patient migration to receive CA from other regions (Table S1). The 90 hospitals, 553 beds in average, are members of a voluntary research group for health‐care management and play a role as a critical care center in each region. Data to calculate the rates of PCI for angina and acute MI were also derived from 2018 DPC data. The DPC codes specifying angina and MI were 050050 and 050030, respectively.

DPC data were submitted by all 4764 acute care hospitals with 616 367 beds in 2018 across Japan. Of these acute care hospitals, 593 hospitals provided more than 10 CAs to patients with tachyarrhythmia in 2018.

The study was approved by the ethics committee of Chiba University, School of Medicine.

### 2.2 Statistical analysis

The numbers of CAs per hospital were summarized from 2009 to 2019; the rates of CA per 100 000 population were calculated for all 47 prefectures, based on the address of the DPC hospitals. To adjust for the age distribution of each prefecture, the number of CAs in each prefecture was divided by the prefecture's population aged ≥40 years because most of the patients receiving this procedure were over 40 years old. The wide regional variation was observed in the use of PCI for angina and acute MI in our previous study.^2^ Therefore, as references, the PCI rates for angina and acute MI were calculated in much the same way, and the results were compared with the CA rate via a correlation coefficient. Patient migration was investigated by comparing the patients' address reported in the Patient Summary with the hospitals' address.

A linear regression model was developed to assess factors describing the regional medical supply supporting the use of CA for each prefecture. These factors included the number of arrhythmia specialists reported by the Japanese Heart Rhythm Society,^13^ which was surrogate for the source of local supplies of CA and was described as per 100 000 population aged ≥40 years for each prefecture. The percentage of CAs performed in private hospitals was also used as a factor in describing the type of supply in the region. Additionally, the PCI rates in angina and acute MI were included in the model. A *P*‐value less than .05 was considered to indicate statistical significance.

## 3 RESULTS

**FIGURE 1:**
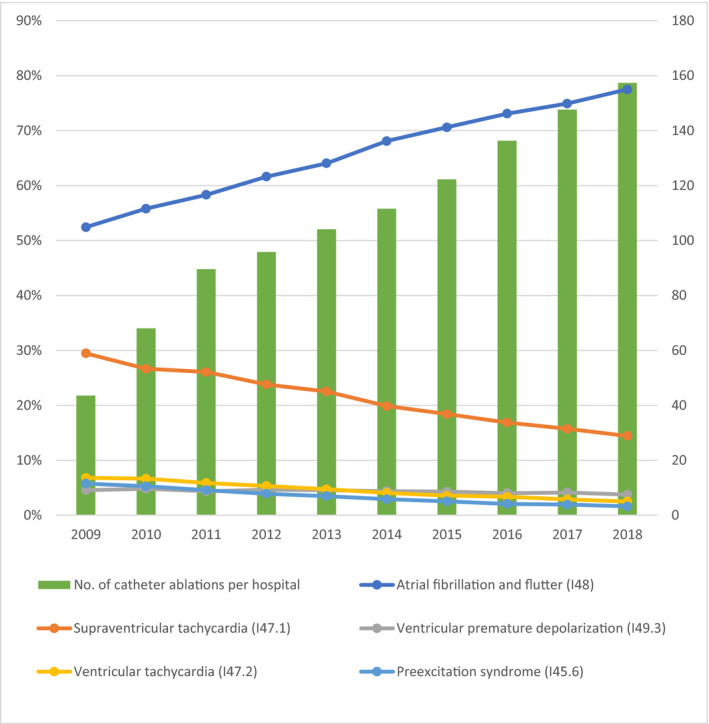
The number of catheter ablations per hospital and the proportion of underlying disease to conduct catheter ablation

A total of 10 972 CAs were performed by 252 DPC hospitals in 2009; as presented in Figure 1, the number of CAs per hospital consistently increased to 2018, where 93 342 CAs were performed by 593 DPC hospitals in Japan. The proportion of underlying disease to conduct CA in 2018 was 78% for AF and atrial flutter, 14% for supraventricular tachycardia, 3% for ventricular tachycardia, 4% for ventricular premature depolarization, and 2% for preexcitation syndrome. The proportion of AF and atrial flutter increased remarkably while the proportion of the rest of the disease was relatively same or slightly decreased.

**FIGURE 2:**
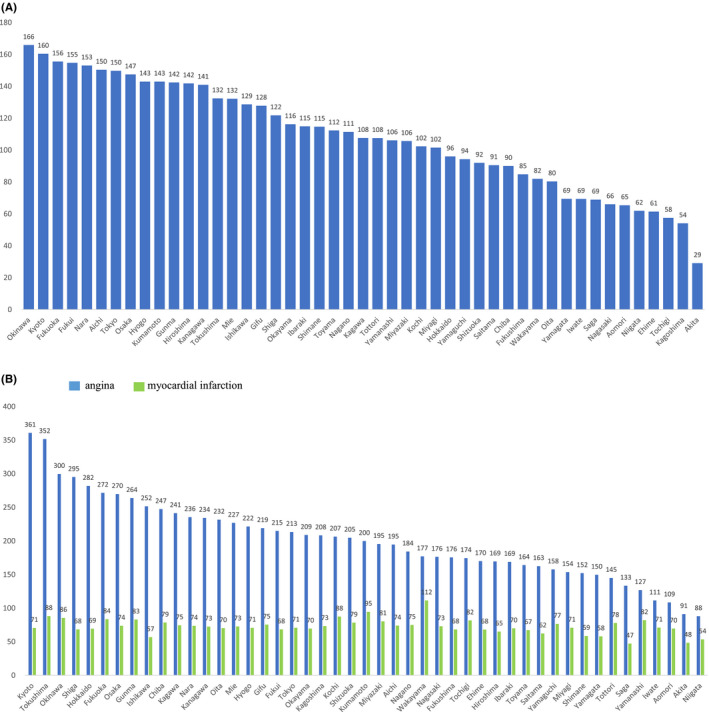
(A) Catheter ablation rate per 100 000 population aged ≥40 years in each prefecture. (B) Percutaneous coronary intervention rate per 100 000 population aged ≥40 years in each prefecture for angina and myocardial infarction

The mean rate of CA in the 47 prefectures in 2018 was 119 per 100 000 population aged ≥40 years. The CA rates by each prefecture are presented in Figure 2. The highest CA rate was 166 in Okinawa prefecture, and the lowest CA rate was 29 in Akita prefecture, which was about half of the second lowest rate (54 in Kagoshima prefecture). The difference between the highest and lowest rate was 5.7‐fold and the difference between the highest and the second lowest rate was still 3.4‐fold.

The highest and lowest PCI rates for angina per 100 000 population aged ≥40 years were 361 and 88, respectively, while those for MI were 112 and 47. The difference between the highest and lowest PCI rates in angina and MI was 4.1‐ and 2.4‐fold. The CA rates and the PCI rates in angina were modestly correlated with each other (*R* ^2^ = .41, *P* < .0001), whereas the CA rates and the PCI rates in acute MI were not associated (*R* ^2^ = .08, *P* = .053). The research group of 90 hospitals provided 20 043 CAs from April 2017 to March 2019. Of them, only 1240 patients (6%) migrated from other prefectures to receive CAs in those hospitals. More than 90% of patients were from same prefecture in 79 of 90 hospitals.

As presented in Table 1, the number of arrhythmia specialists per 100 000 population aged ≥40 years and the PCI rates in angina were significant factors for influencing the use of CA. The more these factors increased, the more CA was performed in each prefecture. On the other hand, other factors were not significantly associated with the use of CA for arrhythmia.

**TABLE 1:** Standardized regression coefficients associated with the catheter ablation rate

Variables	Standardized regression coefficients	*P*‐value
No. of specialists of arrhythmia per 100 000	0.371	.001
No. of PCI for angina per 100 000	0.505	.000
No. of PCI for acute MI per 100 000	0.138	.218
% of CA performed in private hospitals	0.142	.189

Abbreviations: CA, catheter ablation; MI, myocardial infarction; PCI, percutaneous coronary intervention.

## 4 DISCUSSION

A wide regional variation in the use of CA for arrhythmia was observed across Japan. The magnitude of this regional variation was 5.7‐fold between the lowest and highest regions and 3.4‐fold between the highest and second lowest regions across Japan, which was similar to the one in the use with PCI for angina patients, 4.1‐fold in the present study. It was also observed that most of the patients received their CAs in the same regions they lived. From the regression model, the supply of health service resources, that is, the number of arrhythmia specialists, was significantly associated with regional variation.

The DPC data showed a nearly 2.4‐fold increase in the performance of CA per hospital from 2009 to 2018, especially for patients with AF and atrial flutter (Figure 1). Randomized controlled trials conducted in the US and Europe suggested that CA was more effective than antiarrhythmic drug therapy for patients with paroxysmal AF as first treatment.^14^, ^15^, ^16^ The current Japanese guideline gives class I recommendation for patients with symptomatic paroxysmal AF to undergo CAs when antiarrhythmic drug therapy fails.^17^ Corresponding with the evidence and the guideline, the recent result from the Japanese Catheter Ablation Registry for Atrial Fibrillation survey reports a significant increase in the first‐line use of CA, as well as application to patients with a low frequency of AF attacks.^18^ As indicated, a specialist is thought to be a key factor for regional variation. The more specialists who adopt CA as a treatment option in their clinical practice to follow the recent evidence, the larger the regional variation will be that is generated between regions with few specialists and more specialists. When a region has a larger number of specialists, it is likely that a high‐volume center exists in the region, which would also be a large resource for other cardiac procedures, especially for elective procedures. Therefore, the CA rate and the PCI rate for patients with angina would have some correlation with each other but not with the PCI rate for patients with acute MI.

There could be several social reasons as well as the scientific and medical principles for regional variation. Wennberg and colleagues suggested three categories of inappropriate use in health care as reasons of regional variation: underuse of effective care, misuse of preference‐sensitive care, and overuse of supply sensitive care.^19^, ^20^ Underuse of effective care means that the care supported by scientific evidence, such as a vaccine, is not sufficiently provided in the regions; misuse of preference‐sensitive care is when more than one acceptable treatment is available, and the treatment decision is strongly influenced by professional opinion, not by the patients' value; overuse of supply sensitive care occurs when the use of clinical activities, such as hospital admissions, depends on the supply of hospital beds in the regions.^19^ A specialist is likely to be more aware of the recent evidence for the treatment of AF than general practitioners are. This phenomenon would possibly induce preference‐sensitive care based on their professional belief. Japan's universal health insurance system may also encourage them to select CA as a treatment because patient's out of pocket cost is relatively limited. When a new technology becomes available, hospitals usually need some financial investments for the technology. This may be a source for supply sensitive care. Finally, but not less importantly, underuse of effective care could possibly occur in the regions with fewer resources including both human resource and technical resource. Considering our results, reasons for regional variation would be combinations of these inappropriate uses of health care.

Several limitations exist in the present study. According to the survey conducted by the Japanese Circulation Society, the prevalence of AF is different across regions, which also results in regional variation in CA. Thus, the rate of CA was adjusted by population aged ≥40 years to partially mitigate the difference in the prevalence of AF because of aging. Our data were summarized based on the address of the hospital and not the patients, but our data also showed that patients' migration was limited. The assessment reports do not include hospitals with CA performances less than 10 cases per year because of data privacy. In some regions, there exists these small volume hospitals, which may cause or extend the regional variation. Additionally, the patient clinical characteristics were also not available in the assessment report, which may be associated with the regional variation in the CA rate.

In conclusion, the performance of CA has increased over the past 10 years; wide regional variation was observed in the use of CA for patients with arrhythmia in Japan. The study also suggested that regional variation was observed in elective procedures as similar regional variation was observed in the use of PCI for angina patients. Most of the patients underwent CA in hospitals within the same region they lived. As patients with arrhythmia will increase as aging population is growing. Further research is needed to generate evidence of the use of CA for decision‐making as a treatment option and to appropriately deploy this health service regardless of where patients live.

## DISCLOSURE

Authors declare no conflict of interests for this article.

## Supporting information

Supplementary Material
