# Supplementary material for: Regional variation in the use of catheter ablation for patients with arrhythmia in Japan
Source: J Arrhythm. 2020 Nov 21;37(1):22–7. doi: 10.1002/joa3.12455 (PMC7896467; doi:10.1002/joa3.12455)
Supplement: Supplementary file 1 — Supplementary Material [file JOA3-37-22-s001.docx]

Table S1. Location of the research group of 90 hospitals

| Hokkaido and Tohoku | Hokkaido (2), Miyagi (1), Yamagata (1), Akita (1), |
| --- | --- |
| Kanto | Tokyo (9), Saitama (8), Chiba (8), Kanagawa (4), Ibaraki (2), Gunma (1), |
| Tokai and Hokuriku | Aichi (9), Nagano (7), Shizuoka (5), Toyama (3), Gifu (1), Ishikawa (1), Fukui (1), |
| Kinki | Mie (3), Kyoto (2), Shiga (2), Osaka (2), Hyogo (1), Wakayama (1), |
| Chugoku and Sikoku | Hiroshima (2), Okayama (1), Tokushima (1), |
| Kyusyu and Okinawa | Nagasaki (7), Kumamoto (1), Saga (1), Okinawa (2) |

( ) number of hospitals in each prefecture
